# Older and younger job seekers’ attention towards metastereotypes in job ads

**DOI:** 10.1371/journal.pone.0312323

**Published:** 2024-10-30

**Authors:** Aylin Koçak, Nicolas Dirix, Wouter Duyck, Maaike Schellaert, Eva Derous

**Affiliations:** 1 Department of Work, Organization and Society, Vocational and Personnel Psychology Lab, Ghent University, Ghent, Belgium; 2 Department of Experimental Psychology, Ghent University, Ghent, Belgium; Vrije Universiteit Brussel, BELGIUM

## Abstract

Building on social identity theory and cognitive models on information processing, the present paper considered whether and how stereotyped information in job ads impairs older/younger job seekers’ job attraction. Two eye-tracking experiments with older (Study 1) and younger job seekers (Study 2) investigated effects of negatively metastereotyped personality requirements (i.e., traits) on job attraction and whether attention to and memory for negative information mediated these effects. Within-participants analyses showed for both older and younger job seekers that job attraction was lower when ads included negative metastereotypes and that more attention was allocated towards these negative metastereotypes. Older, but not younger job seekers, also better recalled these negative metastereotypes compared to not negative metastereotypes. The effect of metastereotypes on job attraction was not mediated by attention or recall of information. Organizations should therefore avoid negative metastereotypes in job ads that may capture older/younger job seekers’ attention and lower job attraction.

## Introduction

Despite an ongoing ‘war for talent’ [1, 2], qualified older and younger job seekers still experience more difficulties entering the labor market compared to their prime-aged counterparts. Indeed, recent studies report hiring discrimination against older and younger candidates [3–5]. Whereas research preliminary focuses on this age discrimination in hiring (i.e., *select-out*), job seekers’ *self-select out* of application procedures is considered to a smaller extent. That is, job seekers might refrain from applying on the basis of stigmatizing information in job advertisements [6, 7]. The present study investigates effects of stigmatizing information in job ads on older and younger job seekers’ job attraction and hence focuses on job seekers’ own attitudes and experiences [8, 9]. Based on social identity theory, for instance, it is expected that job ads can attract job seekers when the content of job ads indicates a fit between the organization and job seekers’ own social identity [10], that is, the identity that refers to one’s social group (e.g., being female, being older, being younger). If job ads contain age-related cues, this might differently attract older or younger job seekers. However, information in job ads might also capture job seekers’ attention in a *negative* way and lower their attraction to the advertised job. Surprisingly, this has been investigated to a lesser extent and is considered here. Imagine, for instance, a job ad that includes ‘flexible’ in the personality requirements section. Older job seekers might attribute more attention to those traits in job ads that they think others (like recruiters) have negative stereotypes about. When reading ‘we are looking for *flexible* candidates’, older job seekers might believe that others think that older workers are *not flexible*. Similarly, younger job seekers’ attention might be captured by traits such as ‘punctual’, when they believe that others think younger workers are not punctual. These negative stereotypes that group members think out-group members hold about them, or ‘metastereotypes’ [11], might negatively affect job seekers’ job attraction [6, 7] by signaling that the job/organization will not fit their social identity. Therefore, as a first goal, we investigated whether negatively metastereotyped personality requirements (i.e., traits) in job ads are less attractive for older and younger job seekers than not negatively metastereotyped personality requirements.

While Wille and Derous [6, 7] showed that negative metastereotypes in job ads lower ethnic minority and female job seekers’ attraction, we considered older and younger job seekers and additionally investigated the underlying attentional processes that have–to the best of our knowledge–not been considered before. Typically, negative and threatening information captures a reader’s early attention more [12] and is better recalled [13] than non-threatening information. Hence and based on social identity theory [10], we investigated whether negatively metastereotyped personality requirements in job ads might capture job seekers’ attention more and whether they are recalled better than not negatively metastereotyped personality requirements in job ads. As a second goal, and answering a call for more research on underlying mechanisms [6], we not only investigated whether, but also *how* negative metastereotypes in job ads affect older/younger job seekers’ job attraction by investigating whether this effect is mediated by job seekers’ attention and recall. In two eye-tracking experiments, we studied visual attention patterns towards (not) negatively metastereotyped personality requirements in job ads for older (i.e., aged 50–65; Study 1) and younger job seekers (i.e., aged 18–30; Study 2). Below we first discuss effects of metastereotyped information on job seekers’ job attraction, followed by a discussion on the underlying cognitive mechanisms.

## Metastereotypes in job ads

Stereotypes are defined as beliefs about the characteristics of members of a certain group [14]. Age stereotypes, for example, include the idea that younger people are typically more irresponsible and lazy, while older people are typically less flexible and less agreeable [15–17]. Interestingly, older and younger people might be *aware* of these negative age stereotypes and might believe that other people hold these about their own age group. This is referred to as metastereotypes, or “beliefs regarding the stereotype that out-group members hold about his or her own group” [11, p. 917]. For instance, research showed that older workers believed that younger workers find them stubborn, while younger workers believed that older workers find them irresponsible [17]. Note that these cognitions can shape individuals’ attitudes towards and interactions with out-group members, regardless of whether they are true or not.

During recruitment procedures, job seekers can activate negative age-related metastereotypes about information in job ads, for instance, the personality requirements, which can make job seekers’ social category (in this case: age group) more salient. Hence, one may become more aware of the social age group one belongs to (e.g., older job seekers/younger job seekers) and one may perceive oneself more in terms of their social identity (i.e., their social group and related stereotypes), instead of their personal identity (i.e., their own skills, personality, etc…). This social identity [10] is important for job seekers during the recruitment process. When reading job ads, job seekers use the limited information in job ads as cues to evaluate whether the job will fit their social identity, which may hence affect job attraction [18]. Indeed, according to the symbolic attraction theory [18], information that triggers job seekers’ social identity activates a process of making ‘symbolic inferences’ in which job seekers determine whether the job will either fit their social identity or threaten it. Subsequently, job seekers’ job attraction will be higher or lower, respectively.

Thus, when job ads activate age-related metastereotypes that are *negative* in nature, this might pose a threat to older and younger job seekers’ social age identity [19] and impact whether they intent to apply for the job [20]. Research indeed showed that negative metastereotypes in job ads lowered job attraction compared to job ads without negative metastereotypes for female job seekers [7] and ethnic minority job seekers [6]. Similarly, for older and younger job seekers who were shown to each hold specific negative age metastereotypes related to their own age group [17], we expected based on the social identity theory [10] that:

**Hypothesis 1.** Job seekers’ job attraction is lower for job ads with negatively metastereotyped traits than for those without negatively metastereotyped traits.

## Early attention bias

Research showed that people have a vigilance for cues that are negative or threatening [21], meaning that *early* in one’s cognitive processing of information, there might be an attention/detection bias towards negative, threatening information [i.e., ‘early attention’; 22]. Evidence for this attention bias–stemming from the historic survival value of threatening information [23]–was shown for different types of threatening cues, such as: pictures of death/suffering [21], pain cues [24], angry faces [25, 26], threatening animals [27, 28], and even visual stimuli that signal an aversive white noise [29]. Interestingly, vigilance for negative information has also been shown for threats to one’s social identity, for instance for words that activated sexism for women [12]. Since research showed that negative age-related metastereotypes might threaten job seekers’ social age identity [19, 30], the present study aimed to investigate whether this attention bias towards social identity threatening words might occur among older/younger job seekers in a recruitment context.

## Early attention to metastereotypes in job ads

Studies have reported that stigmatizing information (e.g., facial stigma) has an attention-grabbing effect for recruiters during the interview stage and can hence hurt candidates’ chances [31, 32]. In the current study, we investigated whether *job seekers’* attention towards stereotyped cues in stages prior to the interview stage, namely the recruitment stage in which job seekers read job ads, can hinder their chances through self-selection processes [6, 7, 33]. That is, negatively metastereotyped traits portrayed in job ads might act as cues that pose a threat to job seekers’ social identity and capture job seekers’ attention in a negative way, which may lower job attraction. Indeed, previous studies support this signaling purpose of cues during recruitment [6, 7, 34, 35] but did not directly measure the cognitive, attention processes that underlie these effects [6]. Studies did touch on the idea the amount of attention that certain job ad components receive affects attitudes towards the job (ad). For instance, Barber and Roehling [36] employed a thinking-aloud interview method to investigate how applicants process job ad information while reading job ads and how this effected their decisions to apply for the job ad. Such self-report may of course induce demand characteristics, decrease external validity, and participants may also not always be aware of their unconscious mental processes. More recently, Pfiffelman et al. [37] used *eye-tracking* methodology to investigate job seekers’ attentional patterns towards information in job ads that is perceived as negative, as well as the consequential effect on attitudes towards the job. More specifically, they found that personalized LinkedIn job ads (i.e., including job seeker’s name and LinkedIn picture) captured job seekers’ attention, which negatively affected attitudes towards the job ad through perceived intrusiveness of job ad information. In line with these studies, we expect that visual attention to negative job ad information may be associated with more negative attitudes towards the job. Hence, we expected more visual attention towards negative/threatening information in the job ad to lower job attraction for job seekers. Taken together, we expected for older and younger job seekers:

**Hypothesis 2.** Job seekers will allocate more early attention towards negatively metastereotyped traits in job ads compared to not negatively metastereotyped traits, which will hence lower job attraction for job ads with negatively metastereotyped traits.

## Information recall

Stereotyped cues that pose a threat to one’s social identity might not only capture individuals’ attention, they might also impair one’s cognitive functioning [38], like working memory. For instance, information recall levels of older-aged people [39] as well as younger-aged people [40] can be impaired when confronted with cues that activate negative age stereotypes (i.e., task instructions or explicit statements that imply that older/younger people tend to perform worse). Building on self-regulation theory [41], dealing with negatively stereotyped information requires more self-regulating processes and hence may deplete cognitive resources that are needed for working memory tasks, such as information recall (i.e., of new, *non-threatening* information; [42] For instance, Johns et al. [43] found that inducing gender-threatening cues to the experimental lab setting decreased women’s performance on a reading-span task in which female participants were presented with (non-threatening) words that they were asked to recall. In a study of Buijsrogge et al. [32] in a job interview context, interviewers’ recall of general, non-threatening interview content (e.g., candidate information like work experiences) was impaired when interviewers were presented with candidates with visual stigma (like a port-wine stain). In the present study, we investigated recall of not only the neutral/non-threatening job ad information, but also the *threatening* information in job ads (i.e., the negatively metastereotyped traits) itself. That way, we aimed to directly compare job seekers’ memory for stereotyped versus non-stereotyped information in job ads. Similarly, Kanar et al. [13] showed that negative information about the job/organization (i.e., transferred through word of mouth or a business press articles) was better recalled by job seekers than positive information during the pre-hiring stage.

Kanar et al. [13] did not consider the effects of the discrepancy in information recall between negative and positive job information on attitudes towards the organization, such as attraction. However, according to the memory-for-facts model [44], information that individuals can recall (e.g., about advertisements) does affect their attitudes. Yet, over the years, scholars found that the relationship between information recall and attitudes might depend on the exact reading or processing task and should therefore be investigated in a multitude of contexts/situations to further establish the boundary conditions of this relationship [45–49]. Addressing this call, the present study studied the relationship between recall and job attraction in the context of job advertisements. While studies have indeed linked working memory processing of job ad information to job seekers’ attitudes to the organization [8], this has been done in a more indirect way. For instance, job ad information with a higher level of specificity led to higher attraction to the organization, because more specific information is assumed to generate a more elaborate cognitive processing [50]. However, this assumption regarding underlying working memory processes was not empirically tested. The present study aims to investigate information processing in job ads in a more direct way, through measuring older/younger job seekers’ recall of negatively metastereotyped personality requirements in job ads. We hence expected for older and younger job seekers:

**Hypothesis 3.** Job seekers will better recall negatively metastereotyped traits in job ads compared to not negatively metastereotyped traits, which will hence lower job attraction for job ads with negatively metastereotyped traits.

## From early attention to recall to job attraction

While we expect that negative metastereotypes will receive more early attention and will be better recalled by job seekers, it is also expected that more attention towards negative metastereotypes will be related to a better recall of those negative metastereotypes. That is, building on Baddeley and Hitch [51]’s working memory model, ample evidence has shown that more visual attention to a certain location leads to a better transfer of information on that location into the working memory [52–54]. Negatively metastereotyped traits in job ads that are expected to capture older/younger job seekers’ early visual attention more, might therefore also be better recalled by older/younger job seekers than not negatively metastereotyped traits in job ads. Taken together, since both attention and recall are expected to be mediators in the relationship between type of traits (negatively metastereotyped or not) and job attraction and attention is expected to–in its turn–affect recall, we expected the following serial mediation model to explain why negatively metastereotyped traits lower job attraction for older and younger job seekers:

**Hypothesis 4.** Job seekers will allocate more early attention towards negatively metastereotyped traits in job ads compared to not negatively metastereotyped traits, which will hence increase recall of negatively metastereotyped traits in job ads and in turn, lower job attraction for job ads with negatively metastereotyped traits.

**Fig 1:**
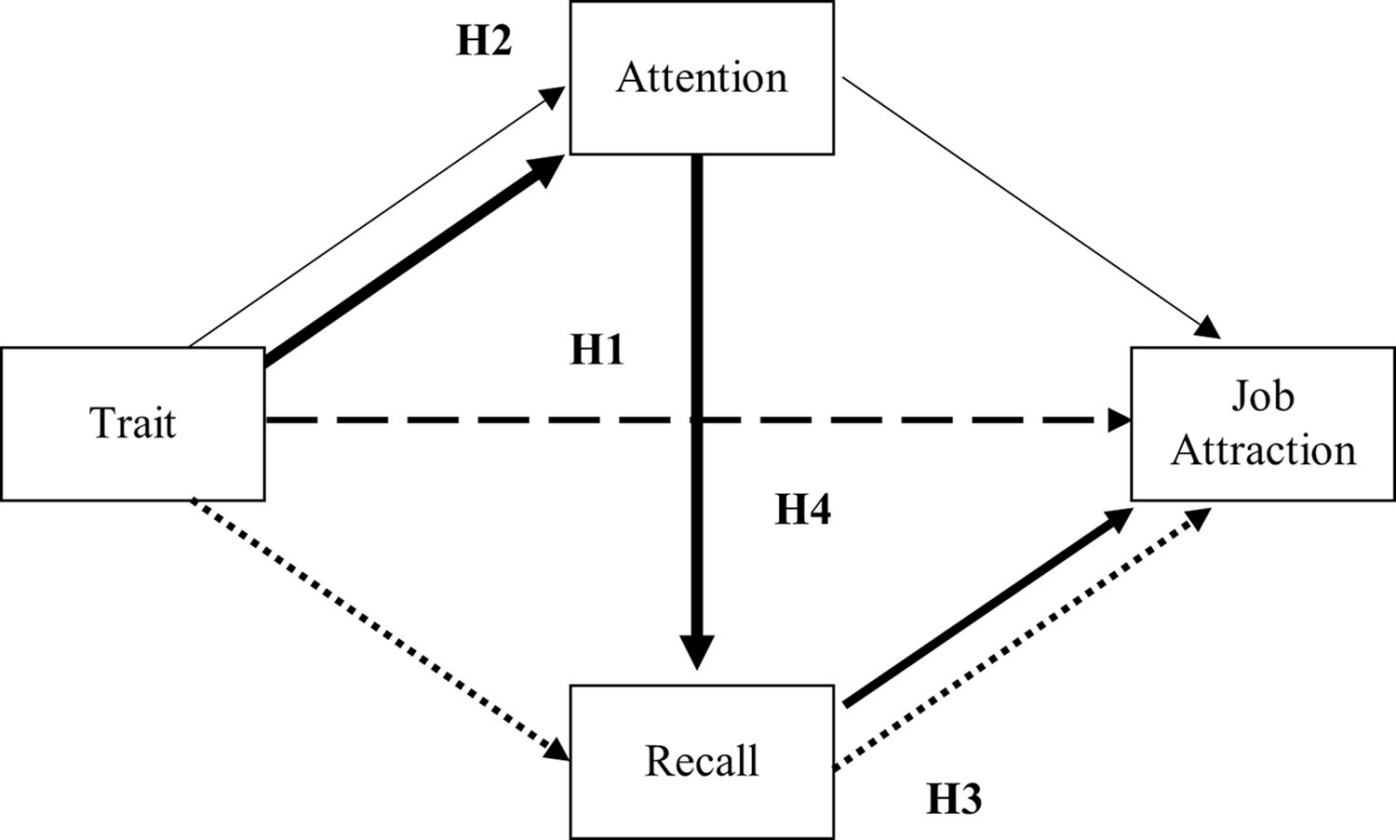
Diagram of Hypotheses 1–4. *Note*. H1 (striped line) investigates the effect of Trait on Job Attraction (total effect). H2 (thin lines) investigates the effect of Trait on Job Attraction via Attention (first mediation). H3 (dotted lines) investigates the effect of Trait on Job Attraction via Recall (second mediation). H4 (bold lines) investigates the effect of Trait on Job Attraction via Attention and Recall (serial mediation).

To test the hypotheses we conducted two eye-tracking experiments. While different methods and tasks can be used to measure one’s attention to stimuli [e.g., attentional search task; Posner cueing tasks and modifications; 29], the current study measured participants’ eye-movements by means of eye-tracking technology, which is often used in a marketing context to study people’s visual attention towards information in advertisements, as well as in more fundamental research on reading tasks [55, 56]. In eye-movement research, a distinction is made between fixations (i.e., when the eyes remain stationary) and saccades (i.e., the fast movement from one fixation point to the next). During the fixations, information from the visual field is extracted, so a tight link between fixations and the locus of attention is presumed [56, 57]. An important advantage of eye-tracking is that it allows for a detailed spatial and temporal measurement of eye-movements, while people perform tasks that are highly similar to their daily life counterparts (e.g., reading job ads), so that the ecological validity of the method is high. Since research showed that both older and younger people might experience threat when being confronted with negative cues and hence experience consequences for their cognitive processing [39, 40], we tested identical paths for both older (Study 1) and younger (Study 2) job seekers. However, as the content of the metastereotypes differs for older and younger job seekers [17], we conducted two separate experiments in which we used negative metastereotypes that are specific for either older or younger job workers, as further explained in Studies 1 and 2. Fig 1 presents diagrams with the hypotheses of Study 1 and Study 2.

## Study 1

Study 1 investigated whether *older* job seekers allocate more early attention to, better recall and are less attracted to negatively metastereotyped traits in job ads, compared to not negatively metastereotyped requirements as well as mediating effects of attention and recall. Older participants were aged 50–65 years, based on McCarthy et al. [58] who found that managers typically consider someone an ‘older’ worker when they are aged 50 or older and research that established that people older than 50 experience specific metastereotypes and discrimination from that age on [17, 59].

### Method of Study 1

#### Participants

A total of 54 older job seekers (ranging from 50 until 65 years old, *M*_age_ = 54.74 years, *SD*_age_ = 3.43; 66.7% women; 100% White/Caucasian ethnicity) were recruited (between September 1^st^ 2020 and August 31^st^ 2021) via professional networks (i.e., via official social media accounts of the research consortium) and snowballing method. Participants received financial compensation (i.e., €10) for their participation in the study.

#### Design and measures

An eye-tracking experiment was conducted that featured a two-condition within-participants design, in which personality requirements in job ads (*trait*: negative metastereotypes vs. not negative metastereotypes; see paragraph ‘Stimuli’ for examples) were manipulated and job attraction, attention and recall were the outcome variables. *Job attraction* was measured after each job ad with three items based on Van Hooft et al. [60], e.g., “I am attracted to the advertised job”, on 5-point Likert scale with 1 = *strongly disagree* to 5 = *strongly agree*. Cronbach’s alpha for the items ranged from .94 to .97 in the condition with negative metastereotype (*M*_cronbach’s alpha_ = .96) and .88 to .97 in the condition without negative metastereotype (*M*_cronbach’s alpha_ = .94). In order to measure *early visual attention* to traits in job ads, compared to early visual attention to other job ad information, we divided study materials (i.e., job ads) into seven interest areas and investigated visual attention towards each of these areas by means of eye-tracking (i.e., eye fixations, see below). More specifically, to measure participants’ early attention to the profiles, we measured their *first run dwell time* [22], i.e., the sum of the duration (in milliseconds) of all fixations within the interest area of the profile during participants’ first pass through the job ad, and compared that to their first run dwell time to the other interest areas. In order to account for job seekers’ visual attention towards the profiles, as well as to the other interest areas of the job ad, we calculated the difference between participants’ first run dwell time to the interest area of the profiles and the average of their first run dwell time to all other interest areas and used this difference score as our early visual attention measure.

To measure *recall* of the traits, we built on Kanar et al. [13]. After reading and rating the job ads, participants were asked to write down the traits that they were able to recall from the profiles in the ads in a two-minute window. Next, manipulation checks were administered to ascertain that our manipulations of the *content* of the traits and their *metastereotyped connotation* were perceived as intended. Example items are “Does the person profile show that they were looking for an *agreeable* or *conscientious* person? [choose one option]”, and “To what extent do you believe that younger workers think that older workers are [obedient / flexible / friendly / patient / compliant]?”, with 1 = *strongly disagree* to 5 = *strongly agree*. Finally, demographic question regarding participants’ age (in years) and gender (0 = *male*; 1 = *female*, *2 = other*) were completed.

#### Stimuli

**Fig 2:**
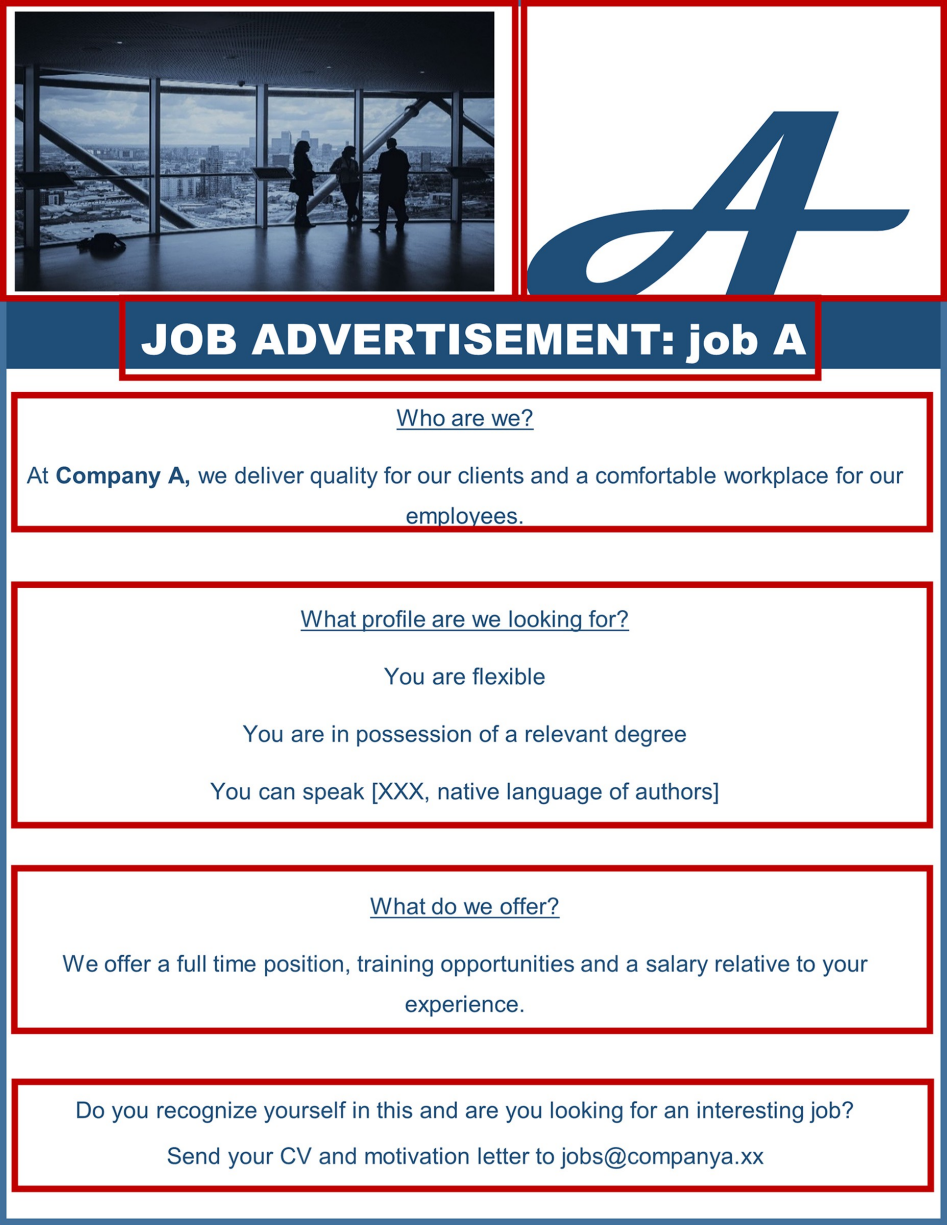
Example of job advertisement with seven interest areas.

Study materials were fictional job advertisements. Building on Hilberink-Schulpen et al. [61], we distinguished the following sections in the job ads (see Fig 2): picture, logo, title, company information, profile with personality requirements (i.e., traits), job offer and contact information. Manipulations were situated in the profile section; profiles contained HEXACO-traits [62, 63] that older job seekers held either negative or no negative metastereotypes about. These negatively metastereotyped and not negatively metastereotyped traits for older people were developed and pilot tested in a previous study of this research project [20]. A more detailed description of the procedure and results of this pilot study can be retrieved from the first author. Results of the pilot study showed that older job seekers hold a negative metastereotypes about the HEXACO-trait Agreeableness, and no negative metastereotype about the HEXACO-trait Conscientiousness. Subsequently, the pilot study revealed the most negatively metastereotyped adjectives “obedient”, “flexible”, “friendly”, “patient”, and “compliant”, which represent the condition with a negative metastereotyped connotation (Agreeableness) and the least negatively metastereotyped adjectives “punctual”, “perfectionistic”, “orderly”, “disciplined”, and “dutiful”, which represent the condition without a negative metastereotype (Conscientiousness). The (not) negatively metastereotyped personality requirement was supplemented with other requirements that were held constant across job ads (i.e., required language proficiency and relevant educational degree for the advertised job). No organization name or type of organization/industry was mentioned *(“Company A”*, *“Company B”)*, as research has suggested that organizational familiarity might affect job seekers’ application intention [64]. Similarly, no job characteristics were mentioned as those characteristics might differentially attract older job seekers [65]. A short company description of the company was held constant across job ads, as well as the offer and contact information. The logo was adjusted based on the letter of the company *“A”* for company A, *“B”* for company B etc. (see Fig 2).

#### Procedure and experimental apparatus

Study 1 was approved (through written consent) by the Ethical Commission of Ghent University in accordance with the Helsinki declaration [Special Ethical Protocol no 2020/77]. At the start of the experiment, participants signed an informed consent (i.e., written consent) and were positioned in front of the eye-tracker. They placed their head in a chin- and headrest to minimize head movements. Once seated, they performed a 9-point calibration procedure. After a successful calibration, participants were instructed to carefully read and evaluate the presented job advertisements. They were also instructed to imagine that the parts of the job ads that were not displayed would suit their interest/qualifications. A total of ten job ads (five for each experimental condition) were presented to participants in a randomized order. After each job ad, participants answered the three items regarding job attraction on the computer screen. On completion of reading all job ads, participants moved away from the eye-tracker and completed the recall question and additional manipulation checks/demographical questions through an online survey on a different computer. Participants’ eye-movements and fixations were measured by means of the Eyelink 1000 (SR Research, Canada; see Table 1) with a spatial resolution of less than 1/4 degrees of visual angle at a sampling rate of 1000Hz. Viewing was binocular, but only the right eye was tracked; Job advertisements were presented on a 1920x1080 Beng XL2411Z LED-monitor at a viewing distance of 95cm with a refresh rate of 144 Hz. Additional to the calibration at the start of the experiment, eye-tracking accuracy was also measured during the experiment by mean of drift checks. When eye-tracking accuracy was low (i.e., higher average error than 0.5˚), the experiment was terminated and data was not included in the analyses.

**Table 1:** Description and performance estimates of EyeLink 1000 tower and desktop mount.

		EyeLink 1000 Tower Mount performance estimates	EyeLink 1000 Desktop Mount performance estimates
Measure			
	Max. Sampling Rate	2000 Hz (Monocular)	2000 Hz (Monocular)
	Tracking principle	Pupil with Corneal Reflection	Pupil with Corneal Reflection
	Accuracy	Down to 0.15°; 0.25˚ – 0.5˚ typical	Down to 0.15°; 0.25˚ – 0.5˚ typical
	Resolution	0.01° RMS, micro-saccade resolution of 0.05°	0.01° RMS, micro-saccade resolution of 0.05°
	Sample Delay	*M* < 1.34 msec, *SD* < .2 msec	*M* < 1.34 msec, *SD* < .2 msec
	Real-Time data	1.4 msec (SD < 0.2 msec) @ 2000 Hz	1.4 msec (SD < 0.2 msec) @ 2000 Hz

### Results of Study 1

#### Preliminary analyses

Table 2 shows descriptives, reliabilities, and correlations among study variables. First, manipulations were successful. Generalized Estimating Equation analysis (SPSS, v26) showed that older participants perceived those traits referring to Conscientiousness significantly more as Conscientiousness (75.9%) than Agreeableness (24.1%), compared to the traits referring to Agreeableness, which were perceived significantly more as Agreeableness (96%) than Conscientiousness (4%), *b* = 4.34, SE = 0.37, Wald χ^2^(1) = 133.00, *p* < .001. Further, results from a repeated measures anova on all adjectives used in the job ads showed that participants believed that younger people find older workers more conscientious (*M* = 3.80, *SD* = 0.59) than agreeable (*M* = 2.92, *SD* = 0.62), *F*(1, 53) = 91.94, *p* < .001, *ŋ*_*p*_*^2^* = .63. Finally, since word frequency might affect how words are processed, i.e., frequency effect; Cop et al. [66], and fixation times, we first investigated word frequency of our stimuli based on Keuleers et al. [67]’s database. No significant difference in word frequency was observed between the condition of negative metastereotypes (*M* = 3.42, *SD* = 0.59) and not negative metastereotypes (*M* = 3.00, *SD* = 0.59), *t*(8) = 1.08, *p* = .31, Cohen’s *d* = .62. This ensures that viewing time differences reflect attention, and not word-level frequency effects.

**Table 2:** Descriptives, internal consistency and correlations of study variables.

	Study 1		Study 2									
	*M*	*SD*	*M*	*SD*	1	2	3	4	5	6	7	8
1. Job Attraction^a^ negative MS^b^	3.00	0.61	2.88	0.48	(.96)/(.88)	.41	-.23	-.16	.02	-.19	.04	-.12
2. Job Attraction not negative MS^b^	3.36	0.64	3.22	0.51	.68**	(.94)/(.91)	.20	.15	.23	.20	.35*	-.14
3. Early attention^c^ negative MS^b^	1316.06	1268.72	1190.59	919.35	-.09	.10	(—)	.54**	.27	.07	-.00	.10
4. Early attention^c^ not negative MS^b^	935.36	1082.28	671.88	540.56	-.09	.09	.70**	(—)	.28	.13	.04	.06
5. Recall^d^ negative MS^b^	2.15	0.94	1.55	1.00	-.16	-.03	.01	.04	(—)	.28	.15	.07
6. Recall^d^ not negative MS^b^	1.59	1.08	1.81	1.05	-.23	-.26	-.09	-.10	.13	(—)	.18	-.09
7. Gender^e^^,^ ^f^	0.67	0.47	0.67	0.47	.33*	.17	.08	.25	.26	.09	(—)	-.41**
8. Age^g^	54.74	3.43	23.67	2.49	-.09	.00	-.00	.02	-.00	-.25	-.13	(—)

*Note*. Results for Study 1 are displayed under the diagonal (*N* = 54); Results for Study 2 are displayed above the diagonal (*N* = 49). Results on the diagonal represent Cronbach’s alfa for older and younger job seekers, respectively (α_old_/α_young_).

^a^ Job Attraction = measured on 5-point Likert scale with 1 = *strongly disagree* to 5 = *strongly agree*.

^b^ MS = Metastereotype.

^c^ Attention = the sum of the duration (in milliseconds) of all fixations within the interest area of the profile during participants’ first pass through the job ad, compared to sum of the duration (in milliseconds) of all fixations within the other interest areas.

^d^ Recall: amount of remembered traits in two-minute window.

^e^ Spearman correlation.

^f^ Gender: 0 = male; 1 = female.

^g^ Age: all job seekers were aged 50–65 years in Study 1 and all job seekers were aged 18–30 years for Study 2.

*p < .05

**p < .01

#### Hypothesis testing

**Fig 3:**
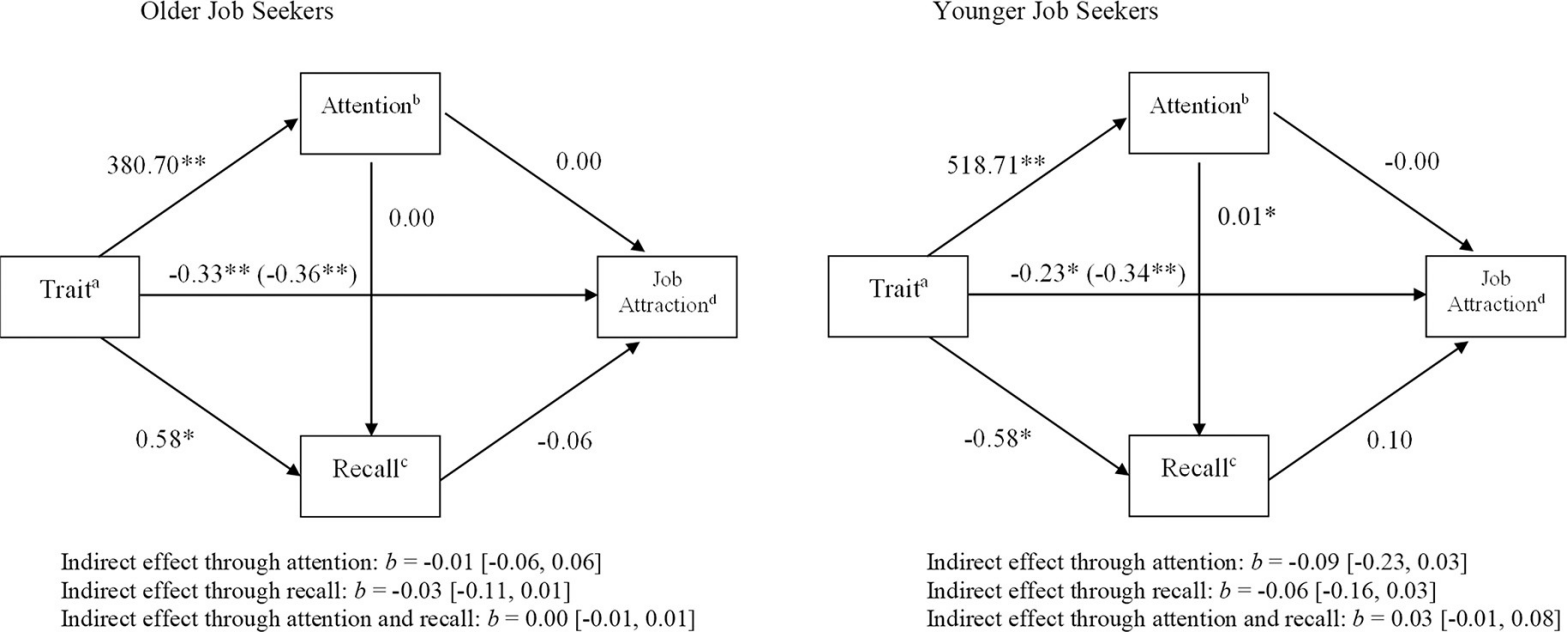
Serial mediation models for older job seekers (Study 1) and younger job seekers (Study 2). *Note*. *N*_Study 1_ = 54; *N*_Study 2_ = 49; Unstandardized coefficients are reported. The coefficients in parentheses represent the total effect of trait on job attraction, i.e., the direct and indirect effects. ^a^Trait 0 = not negatively metastereotyped trait, 1 = negatively metastereotyped trait. ^b^Attention^:^ the sum of the duration (in milliseconds) of all fixations within the interest area of the profile during participants’ first pass through the job ad, compared to sum of the duration (in milliseconds) of all fixations within the other interest areas. ^c^Recall: amount of remembered traits in two-minute window. ^d^ Job Attraction = measured on 5-point Likert scale with 1 = *strongly disagree* to 5 = *strongly agree*. *p < .05. **p < .01.

A within-participant mediation analysis through path analysis [68] using the MEMORE macro [V2.1; 69] was performed to test Hypotheses 1 to 4. This allowed us to test the serial mediation model with attention and recall as mediators and report difference scores between the condition with and without negative metastereotype. Results are displayed in Fig 3.

First, results showed that older job seekers were significantly less attracted to jobs when the job ad included negatively metastereotyped traits compared to when they included not negatively metastereotyped traits, *b* = -0.36, *SE* = 0.07, *t*(53) = -5.30, *p* < .001, supporting Hypothesis 1.

Next, Hypothesis 2 expected that early attention mediates the relationship between type of trait and job attraction for older job seekers. Although older job seekers indeed allocated 40.7% more early attention to negatively metastereotyped traits in job ads compared to not negatively metastereotyped traits, *b* = 380.70, *SE* = 129.47, *t*(53) = 2.94, *p* < .001, early attention towards negatively metastereotyped traits in job ads did not significantly relate to lower job attraction, *b* = 0.00, *SE* = 0.00, *t*(49) = -0.20, *p* = .84. Moreover, the indirect effect of type of trait on job attraction through early attention was not significant, *b* = -0.01, bootstrapped *SE* = 0.03, bootstrapped 95% CI = [-0.06, 0.06]. Hence, Hypothesis 2 could not be supported for older job seekers.

Further, Hypothesis 3 predicted that recall would mediate the relationship between type of trait and job attraction. Results showed that, in line with expectations, older job seekers indeed better recalled negatively metastereotyped traits in job ads compared to not negatively metastereotyped traits, *b* = 0.58, *SE* = 0.20, *t*(51) = 2.85, *p* = .01. However, better recall of negatively metastereotyped traits in job ads was not significantly related with lower job attraction, *b* = -0.06, *SE* = 0.05, *t*(49) = -1.11, *p* = .27 and the indirect effect of type of trait on job attraction through recall was also not significant, *b* = -0.03, bootstrapped *SE* = 0.03, bootstrapped 95% CI = [-0.11, 0.01]. Hypothesis 3 could therefore not be supported for older job seekers.

Finally, the serial mediation as predicted by Hypothesis 4 could not be supported for older job seekers. That is, more early attention to negatively metastereotyped traits in job ads was not significantly related with better recall, *b* = 0.00, *SE* = 0.00, *t*(51) = -0.27, *p* = .79 and the indirect effect of type of trait on job attraction through attention *and* recall was also not significant, *b* = 0.00, bootstrapped *SE* = 0.00, bootstrapped 95% CI = [-0.01, 0.01].

### Discussion of Study 1

In line with predictions from social identity theory [10], older job seekers were less attracted to jobs when job ads contained negatively metastereotyped traits, signaling that the job does not fit with their own age-identity. Older job seekers indeed allocated more early visual attention towards negative metastereotypes in job ads [12] and better recalled the negative metastereotypes compared to the not negative metastereotypes [13]. Attention to and recall of negatively metastereotyped traits in job ads were, however, not related to older job seekers’ job attraction. We measured job seekers’ early attention to investigate a *vigilance* for negative metastereotypes in job ads, yet future research might test whether an early attention bias towards negative metastereotypes in job ads is followed by a different attentional pattern in later stages (e.g., avoidance) and is hence not positively related to working memory and job attraction. Contrary to previous expectations rooted in Baddeley and Hitch [51]’s working memory model [e.g., 52–54], more attention to the negative metastereotypes did not increase recall of negative metastereotypes and no mediating effects of either attention or recall were found. Additional, emotional-motivational processes (see general discussion) might explain why no effects were found [e.g., 70] and need to be considered in future research. We proceeded testing Hypotheses 1 until 4 for younger job seekers, using (not) negatively metastereotyped traits for younger people.

## Study 2

Study 2 investigated the same hypotheses as Study 1 and tested whether *younger* job seekers are less attracted to, allocate more early attention to and can better recall negatively metastereotyped traits in job, compared to not negatively metastereotyped traits, as well as the mediating mechanisms of attention and recall. Participants were all aged 18–30 years, based on Arnett [71]’s life stage transition to young adulthood that is situated around the age of 30y and Finkelstein et al. [17] who found specific metastereotypes for people younger than 30 years. The method that was used in Study 2 was identical to the method employed in Study 1, unless explicitly stated otherwise.

### Method of Study 2

#### Participants

A total of 49 younger job seekers (ranging from 18 until 30 years old, *M*_age_ = 23.67 years, *SD*_age_ = 2.50; 67.3% women, 100% White/Caucasian ethnicity) were recruited (between September 1^st^ 2020 and August 31^st^ 2021) through the professional network of the researchers (e.g., social media accounts of the research consortium) and snowballing method. Participants received financial compensation (€10) for their participation in the study.

#### Design and measures

We conducted an eye-tracking experiment among younger job seekers that, identically to Study 1, featured a two-condition within-participants design, in which traits in job ads (*trait*: negative metastereotypes vs. not negative metastereotypes) were manipulated and job attraction, attention and recall were outcome variables. Identical measures were used for job attraction [i.e., three items based on Van Hooft et al. [60], Cronbach’s alpha for the items ranged from .84 to .91 in the condition with negative metastereotype (*M*_cronbach’s alpha_ = .88) and .89 until .95 in the condition without negative metastereotype (*M*_cronbach’s alpha_ = .91)], visual attention [i.e., difference in *first run dwell time* to the interest area and to the other interest areas; 22], recall [i.e., recalled requirements in two-minute window; 13], and demographical questions. Manipulation checks to test the *content* of the traits and their *metastereotyped connotation* were completed. Example items are “Does the person profile show that they were looking for a *conscientious* or *open* person? [choose one option]”, and “To what extent do you believe that older workers think that younger workers are [punctual / perfectionistic / orderly / disciplined / dutiful]?”, with 1 = *strongly disagree* to 5 = *strongly agree*.

#### Stimuli

Similar to Study 1, materials were fictional job advertisements but the manipulation of traits in the profile section was now tailored to younger job seekers: profiles contained traits that younger job seekers held either negative or no negative metastereotypes about. As in Study 1, we developed and pilot tested the traits in a previous study [20]. A more detailed description of the procedure and results of this pilot study can be retrieved from the first author. Results showed that younger people held a negative metastereotype about the HEXACO-trait Conscientiousness and no negative metastereotype about the HEXACO-trait Openness to Experience. Based on the pilot study, we selected “punctual”, “disciplined”, “deliberative”, “consistent”, and “diligent”, for the condition with negative metastereotype (Conscientiousness) and “inventive”, “creative”, “open-minded”, “sharp-witted” and “versatile” for the condition without negative metastereotype (Openness to Experience). Other requirements were held constant across job ads, just as a short company description, the offer and contact information. No organization name, type of organization/industry or job characteristics were mentioned, and as for the logo, we used was an *“A”* for company A, etc. (see Fig 2).

### Procedure and experimental apparatus

Study 2 was approved (through written consent) by the Ethical Commission of Ghent University in accordance with the Helsinki declaration [Special Ethical Protocol no 2020/77]. At the start of the experiment, participants signed an informed consent (i.e., written consent). Both the procedure and the experimental apparatus of Study 2 were identical to that of Study 1 (see above).

### Results of Study 2

#### Preliminary analyses

Table 2 shows descriptives, reliabilities, and correlations among study variables.

Our manipulations were successful: Generalized Estimating Equation analysis showed that younger participants perceived those traits referring to Openness significantly more as Openness (83.1%) than Conscientiousness (16.9%), compared to the traits referring to Conscientiousness, which were perceived significantly more as Conscientiousness (94.5%) than Openness (5.5%), *b* = 4.44, SE = 0.33, Wald χ^2^(1) = 183.76, *p* < .001. Further, repeated measures anova results showed that participants believed that older people find younger workers more open (*M* = 3.98, *SD* = 0.58) than conscientious (*M* = 2.53, *SD* = 0.67), *F*(1, 53) = 91.94, *p* < .001, *ŋ*_*p*_*^2^* = .85. Again, no significant difference was observed in word frequency between the condition of negative metastereotypes (*M* = 2.98, *SD* = 0.40) and not negative metastereotypes (*M* = 2.71, *SD* = 0.89), *t*(8) = .61, *p* = .56, Cohen’s *d* = .69, which excludes low word-level differences between crucial conditions.

#### Hypothesis testing

Similar to Study 1, we performed a within-participant serial mediation analysis through path analysis [68] with the MEMORE macro [V2.1; 69] to investigate Hypotheses 1 to 4. Results are displayed in Fig 3. First, younger job seekers were significantly less attracted to jobs when the job ad included negatively metastereotyped traits compared to not negatively metastereotyped traits, *b* = -0.34, *SE* = 0.08, *t*(48) = -4.37, *p* < .001, supporting Hypothesis 1.

Further, Hypothesis 2 tested the mediating effect of attention between type of trait and job attraction. While younger job seekers indeed allocated 77.2% more early attention to negatively metastereotyped traits in job ads compared to not negatively metastereotyped traits, *b* = 518.71, *SE* = 122.82, *t*(48) = 4.22, *p* < .001, early attention towards negatively metastereotyped traits in job as did not significantly relate to job attraction, *b* = -0.00, *SE* = 0.00, *t*(44) = -1.50, *p* = .14. Moreover, given that the indirect effect of type of trait on job attraction through early attention was not significant, *b* = -0.09, bootstrapped *SE* = 0.07, bootstrapped 95% CI = [-0.23, 0.03], Hypothesis 2 could not be supported for younger job seekers.

Further, Hypothesis 3 investigated the mediating effect of recall between type of trait and job attraction. Contrary to our expectations, younger job seekers better recalled the *not* negatively metastereotyped traits in job ads compared to negatively metastereotyped traits, *b* = -0.58, *SE* = 0.21, *t*(46) = -2.78, *p* = .01. Furthermore, better recall of negatively metastereotyped traits in job ads was not significantly related with job attraction, *b* = 0.10, *SE* = 0.06, *t*(44) = 1.48, *p* = .15. Next, as the indirect effect of type of trait on job attraction through recall was not significant, *b* = -0.06, bootstrapped *SE* = 0.05, bootstrapped 95% CI = [-0.16, 0.03], Hypothesis 3 could not be supported for younger job seekers.

Finally, Hypothesis 4 expected a serial mediation model with attention and recall as serial mediators between type of trait and job attraction. First, more early attention to negatively metastereotyped traits in job ads was indeed related with better recall, *b* = 0.01, *SE* = 0.00, *t*(46) = 2.58, *p* = .01. However, the indirect effect of type of trait on job attraction through attention *and* recall was not significant for younger job seekers, *b* = 0.03, bootstrapped *SE* = 0.02, bootstrapped 95% CI = [-0.01, 0.08], providing no support for Hypothesis 4.

### Discussion of Study 2

Similar to Study 1, Study 2 results showed that younger job seekers’ job attraction was lower for job ads with negatively metastereotyped traits, compared to job ads with not negatively metastereotyped personality requirements. Younger job seekers also allocated more early attention to negatively metastereotyped personality requirements in job ads. These findings are in line with social identity theory [10] and an attention bias towards negative/threatening information [12]. That is, results indicate that negative metastereotypes in job ads might signal to younger job seekers that their social age identity is threatened and hence a lack of fit with the job. However, unlike Study 1 and findings of Kanar et al. [13], no support was found for a better recall of negative metastereotypes in job ads in Study 2. That is, while we expected that negatively metastereotyped traits would be better recalled, the opposite relationship was found and not negatively metastereotyped traits were better recalled (i.e., as marked by the negative regression coefficient in Fig 3). This indicates that the effect of negative metastereotypes on recall might depend on age. As in Study 1, no effects of early attention and recall on job attraction were found and future research initiatives should investigate later or overall attention patterns to negative metastereotypes to provide more insight. Contrary to Study 1, we did find a small positive relationship between attention towards negative metastereotypes in job ads and recall for younger job seekers, in line with predictions from Baddeley and Hitch [51]’s working memory theory and earlier findings. This might be understood in light of the differential working memory performance that has been observed between older and younger people [72]. Finally, no mediating effects of attention and recall on job attraction were found, which might be explained by job seekers’ emotions and motivation, as further discussed below.

## General discussion

Compared to prime-aged people, particularly older (50-65y) and younger (18-30y) people experience specific obstacles when trying to enter the workforce [73, 74]. Despite legislation that prohibits discrimination against people based on their age [75], studies have shown that older and younger job seekers both experience hiring discrimination [4]. Remarkably, studies have overlooked more subtle forms of negative age cues and how they might lead to self-select out in the early stages of the job seeking process. Therefore, the present study investigated whether and how negatively metastereotyped personality requirements in job ads affect older and younger job seekers’ attraction during recruitment procedures.

### Key findings

Previous studies showed that female and ethnic minority job seekers’ job attraction was lower for job ads with negative metastereotypes [6, 7]. The present study built on these results and, as a first goal, investigated these effects among older and younger job seekers. First, results of Study 1 and Study 2 confirmed that job attraction was lower for job ads with negatively metastereotyped traits for both older and younger job seekers. That is, job seekers’ social age identity might also be threatened by negative metastereotypes in job ads and might hence influence job attraction [10, 19].

Further, in terms of our second goal regarding the processes underlying the effect of negative metastereotypes in job ads on job attraction, results of two experimental eye-tracking studies showed that both older and younger job seekers allocated more early visual attention to negative metastereotypes in job ads and provide support for the attention bias towards negative/threatening cues that has been shown in previous studies [21, 24, 29]. Interestingly, the present study showed that this attention bias does not only exist for more imminent threats, but also for information that is ego-threatening, or a threat to one’s social identity [12]. However, attention did not mediate the relationship between type of trait and job attraction, which is not in line with expectations based on previous findings [36, 37], but might be understood in light of the vigilance-avoidance hypothesis. That is, studies have shown that a vigilance or attention bias towards negative information might be followed by an *avoidance* of that negative information [76, 77]. Hence, an early attention-bias towards negative information might not necessarily always result in a more elaborate procession of that information.

Third, we expected that recall would be a mediator between type of trait and job attraction. Remarkably, the expectation that negatively metastereotyped traits in job ad would be better recalled than not negatively metastereotyped traits in job ads was only supported for older job seekers and not for younger job seekers (where we found a significant but negative relationship between type of trait and recall, see Fig 3), despite successful manipulation checks in both age groups. While not in line with our expectations based on social identity theory and previous findings [39, 40], a study by Hehman and Bugental [78] showed that age stereotypes might also threaten younger people to a lesser extent than older people and hence affect their cognitive performance in a different way, because older and younger people might have different, ‘life-stage specific’ experiences. That is, younger people continuously grow older and thus become closer to the group of the ‘prime-aged’ people. Their status in terms of age-stereotypes will therefore improve, which might alter how they process negative age-stereotypes compared to older people whose age-based status will not improve. Indeed, studies found that younger–and not older–people might experience negative (meta)stereotypes more as a challenge [30, 79]. Relatedly, effects of negative age stereotypes might also play out differently for older-aged versus younger-aged people when considering the general ageism and societal bias aimed more at older-aged people compared to younger-aged people [5, 80]. Moreover, both in Study 1 and Study 2, recall of negative metastereotypes was not related to job attraction, providing no support for a general link between information recall and attitudes based on that information (memory-for-factsmodel; [44]. Indeed, studies showed that the link between information recall and attitudes depends on certain conditions, such as the exact processing task [45–49]. The present results indicate that a better recall of negatively metastereotyped information in job ads might not lead to lower job attraction of older and younger job seekers and hence uncovered one boundary condition of the relationship between recall and attitudes [49].

Finally, contrary to Baddeley and Hitch [51], as well as previous findings [52–54], no serial mediating effect of attention and recall was found for older/younger job seekers. Job seekers’ higher early attention levels to and lower job attraction for negative metastereotypes in job ads indicate that older and younger job seekers’ social identity might be threatened by negative metastereotypes in job ads [30]. However, we did not measure alternative appraisal mechanisms such as challenge/boost, nor did we measure job seekers’ *emotional* experiences (e.g., which emotions job seekers experience when reading negative metastereotypes). This suggests that, although a tight link between fixations and visual attention is presumed [56, 57], the relationship between eye-movements and memory is less straightforward and might depend on one’s age. For instance, given that working memory generally declines with age (see further), the lack of relationship between visual attention and memory among older job seekers might be explained by floor effects of the memory task that specifically challenged older job seekers. Interestingly, we know of two other studies that also found no support for the expected positive relationship between visual attention to textual information and recall [81, 82]. Similar to the relationship between attention and job attraction, the relationship between attention and recall might be different when later attentional stages are considered, hence a vigilance-avoidance pattern might explain our current findings. Moreover, the findings that differed between older and younger job seekers were both related to recall/memory. Indeed, research has vastly shown that individual’s working memory generally declines with age and that older participants might perform differently than younger participants on a memory task [72]. However, while this is true for *general* working memory capacity, the effects of negative metastereotypes on older and younger job seekers’ working memory were not considered before. While research has touched on the idea that negative versus positive cues might affect memory of older and younger people differently [83, 84], results were contradictory and scholars also did not consider cues that are threatening for one’s social identity. We thus contribute to the literature by showing that working memory processing of social identity-threatening information might differ between older and younger job seekers.

### Contributions, limitations, and directions for future research

While age-related stereotypes might influence recruiters’ hiring decisions later in the selection process, age stereotypes might also impair older and younger job seekers’ chances earlier, during recruitment procedures. As a first contribution to the literature, the current study thus considered experiences of job seekers during the early stage of the job search process, i.e., while reading job ads and thereby focused on demographic groups that tend to be overlooked (i.e., older and younger job seekers). Study results show that job advertisements used as tools to attract job seekers might also contain signals that can actually capture job seekers’ attention in a *negative* way and lower their attraction to the advertised job.

Second, previous studies on the effects of negative metastereotypes in job ads have not considered the underlying mechanisms that are at play [6, 7]. The present study adds to the existing literature by studying job attraction, as well as the potential mediating effects of early visual attention and recall, hence aiming to uncover the processing of negatively metastereotyped information compared to other information in job ads among older and younger job seekers. In doing, the present study also adds to the literature on cognitive information processing by testing attentional and recall mechanisms in an applied setting, namely the recruitment context. For instance, studies on the attention bias towards negative information focused on negative information that poses a general/imminent threat. Results of Study 1 and Study 2 add to the limited research that supports the attention bias for more subtle cues that pose a threat to one’s social identity [i.e., ego-threat; 12]. Finally, in both studies, we used job advertisements that were complete and realistic, yet manipulated with thoroughly developed and pilot tested stimuli, adding to both the internal and ecological validity of the present study.

As in any study, limitations and directions for future research should be acknowledged.. First, in terms of the cognitive processing of job ads, negatively metastereotyped traits were less attractive for older/younger job seekers and captured their attention. Yet, the exact mechanism in which attention affects job attraction might depend on additional factors that were not studied in the present study. For instance, job seekers’ emotional-motivational mechanisms were not considered. Finkelstein et al. [85] suggested that negative metastereotypes might elicit both positive emotions (e.g., pride) and negative emotions (e.g., anger, sadness) within older/younger job seekers. Since emotions can affect people’s attention [86], memory [87, 88] and attitudes [89–91], future research could therefore consider not only the appraisal of negative metastereotypes in job ads terms of threat, but also in terms of emotional valence (i.e., whether negative metastereotypes elicit positive or negative emotions). Moreover, the emotional valence of information might also affect older and younger job seekers differently. For instance, working memory performance was mitigated by negative emotions for older, but not for younger people [84, 92]. However, study findings remain contradictory, since different studies report no age difference in working memory reaction towards negative emotions between older and younger people [83]. In conclusion, future research might further compare effects of positive versus negative emotions such as anger, sadness and pride on attention, memory and attitudes between older and younger job seekers. Hence, interactional effects between age and emotions can be investigated. Further, not only job seekers’ emotions, also their motivation might influence the processing of and attraction to job ads [8, 50] and should be considered in the future. That is, while we used complete and realistic job ads, no real jobs were at stake and results of our study might be different/stronger when job seekers were presented with an actual job tailored to their interests, since this might increase their motivation [70].

Second, the present study investigated negative metastereotypes related to trait requirements in job ads. However, metastereotypes may not be restricted to trait requirements but may also exist about skills/competences. Future research, therefore, could investigate skills/competences that one holds (no) negative metastereotype about. Moreover, since age-metastereotypes might also be *positive* in nature [17], potentially boosting effects of positive metastereotypes in job ads might also be studied in the future. Third, future research might include measures on the degree to which one identifies with their age group and the degree to which one is concerned about being evaluated by the out-group, since both of these aspects might affect metastereotype activation within job seekers [6, 93]. Further, the link between attention and recall of information and attitudes based on this information might be influenced by personal factors such as one’s self-perceptions [94]. Indeed, Finkelstein et al. [85] showed that individuals’ core self-evaluations (i.e., CSE; the general belief in oneself) might affect how older people react towards negative metastereotypes. Additional research is needed to investigate the role of CSE or one’s self-perceptions on older and younger job seekers’ processing of negative metastereotypes in job ads. Finally, future research might investigate the more behavioral intentions to apply for the job [20] and might also include metastereotypes regarding middle-aged workers [17].

#### Practical implications

Organizations rarely evaluate how job advertisements are perceived by job seekers, although job ads are used to inform and attract job seekers. Results of Study 1 and Study 2 showed that job advertisements with negatively metastereotyped information might capture older/younger job seekers’ attention and decreases their job attraction. Considering the importance of job attraction for application intentions and behavior [95–97], these seemingly subtle cues in job ads might affect the composition of the applicant pool and hence the success of recruitment [6, 7]. In order to obtain an age-diverse applicant pool, organizations are advised to avoid using traits in job ads that activate negative metastereotypes within older and younger job seekers. This might be particularly useful for those organizations that aim to target older and younger job seekers in their recruitment strategy. Targeted recruitment [98], for instance, is a recruitment strategy that organizations can use to target those specific job seekers that are currently underrepresented in the labor market or in their own organization, e.g., older and younger job seekers. Research on targeted recruitment has overlooked how job seekers from those underrepresented groups perceive required qualifications in job ads [6]. Study findings indicate that when job seekers have negative metastereotypes about those qualifications, targeted recruitment strategies can backfire, and job seekers from underrepresented groups can be discouraged by job advertisements instead.

Organizations can use different sources of information to determine negative age-related metastereotypes for older/younger age groups. First, organizations might do ‘sensitivity check’. That is, older and younger employees (if needed with different ethnic backgrounds) can be consulted and share their experiences on possible metastereotypes, for instance, by means of methodologies such as verbal protocol analysis [36] or a cognitive interview [99].

Second, the present study and previous studies on age metastereotypes [17, 85] report traits that older and younger job seekers might have negative metastereotypes about and can be used as a starting point for organizations. Based on these negative metastereotypes, organizations might thoroughly evaluate job advertisements on potentially metastereotyped information. Additionally, machine learning techniques can be used to facilitate this process [100]. Moreover, a distinction could be made between requirements that are crucial (e.g., ‘must haves’) and those that are less crucial (‘nice to haves’). Indeed, negative metastereotypes related to less crucial person requirements could be eliminated and those related to crucial requirements might benefit from a more positive or behavioral wording [30].

Further, apart from eliminating negative information in job ads that grabs job seekers’ attention, organization might also add information in job ads that signals identity safety instead of threat. Davies et al. [101] for instance, suggest using explicit statements during test-taking that stress that “research shows that the underrepresented group does not perform significantly worse on tests” and could also be used in the recruitment context. Adapting the positioning and lay-out of those statements such that they capture readers’ attention more than the negative information might also be an additional suggestion.

Finally, while most people know of the existence of age stereotypes, age *meta*stereotypes are a less known topic. For organizations and recruiters, the existence, content and effects of age metastereotypes can be included in diversity trainings [102]. For job seekers, metastereotype awareness can be provided during career counseling by job coaches.

## Conclusion

Two experimental eye-tracking studies showed that negatively metastereotyped traits captured older/job seekers attention and decreased job attractivity compared to not negatively metastereotyped traits in job ads. Older but not younger job seekers also better recalled these negative metastereotypes compared to not negative metastereotypes. These findings provides unique insight into older/younger job seekers’ processing of negative recruitment information and showed that subtle, but negative cues in job advertisements might have an attention-grabbing effect and lower job attraction of certain groups of job seekers.
